# A comparison of maxillary sinus diameters in Chinese and Yemeni patients with skeletal malocclusion

**DOI:** 10.1186/s12903-022-02633-0

**Published:** 2022-12-09

**Authors:** Abduljabbar Yahya Albarakani, Bo-wen Zheng, Jialin Hong, Majedh Abdo Ali Al-Somairi, Abass Ahmed Abdulqader, Yi Liu

**Affiliations:** 1 Department of Orthodontics, School and Hospital of Stomatology, China Medical University, Liaoning Provincial Key Laboratory of Oral Diseases, Shenyang, 110002 China; 2 Department of Orthodontics and Dentofacial Orthopedics, Faculty of Dentistry, Ibb University, Ibb, Republic of Yemen; 3 Department of Orthodontics, West China School of Stomatology, Sichuan University, Chengdu, Sichuan China

**Keywords:** Cephalometric radiograph, Ethnicity, Gender, Maxillary sinus, Skeletal malocclusion

## Abstract

**Background:**

This study aimed to compare the maxillary sinus dimensions and surface area in accordance with skeletal malocclusion, gender and ethnicity factors in a sample of Chinese and Yemeni patients.

**Methods:**

This cross-sectional study analysed 180 maxillary sinuses using 180 lateral cephalometric radiographs. The patients were subdivided into two ethnic groups: Chinese and Yemeni. Each ethnic group comprised 90 patients, and men and women were divided equally. Each ethnic group was classified into three skeletal classes using ANB and Wits appraisal (skeletal Classes I, II and III). Pearson’s correlation coefficient was also used to assess the relationship between maxillary sinus dimensions and cephalometric parameters.

**Results:**

Men had larger maxillary sinuses than women; skeletal Class II had a higher length and surface area increase than other skeletal classes, although skeletal Classes I and II were almost equal in height. Except for the maxillary sinus length, none of these findings were statistically significant. The maxillary sinuses in Chinese are larger than those in Yemenis (*P* = 0.000). These variables were positively correlated with SNA, SNB and Co–A. The maxillary sinus length and Co–Gn were positively correlated. The NA–APO and NA–FH angles were also correlated with the maxillary sinus surface area. However, the gonial and GoGn–Sn angles negatively affected the maxillary sinus dimension and surface area.

**Conclusions:**

Men had larger maxillary sinuses than women in both ethnic groups, and Chinese individuals had larger maxillary sinuses than Yemenis. Skeletal Class II malocclusion of both ethnicities had larger maxillary sinus dimensions. Furthermore, the maxillary sinus dimensions correlated with cephalometric parameters.

**Supplementary Information:**

The online version contains supplementary material available at 10.1186/s12903-022-02633-0.

## Introduction

The maxillary sinuses consist of two chambers in the upper jawbone filled with air. The sinus apex extends to the zygomatic process, which is contained within the zygomatic bone, alveolar process, first and second molars and the canine roots, which may elevate or perforate the sinus floor. The base of the maxillary sinus is located on the lateral wall of the nasal cavity, whilst its apex extends laterally towards the zygomatic bone. In general, the base of the maxillary sinus is composed of a thin bone plate that surrounds the root ends of the upper posterior teeth. The orbit’s bony floor forms the maxillary sinus’ apex, and their development begins during the third month of pregnancy as an evagination of the lateral wall of the nasal fossa epithelium [1, 2].

Based on the current literature, the maxillary sinus grows postnatally and mostly through the first 3 years of life, between the ages of 7 and 12 [3, 4]. They reach an adult size between the ages of 18 and 20 [5].

Sinuses enter the maxillary alveolar process of most adults and reach the roots of their second premolars and first and second permanent molars. It can sometimes reach the area of the canine root [6]. Therefore, dentists place a high value on the maxillary sinuses because of their proximity to the area they treat, where extractions, implants, endodontic operations and orthodontic mechanics can all be complicated by tooth roots extending into the maxillary sinus [7].

Therefore, this close relationship, along with the upper posterior teeth, is crucial in orthodontic treatment planning [8, 9]. For example, in the absence of the upper first molar, the maxillary sinus may have moved to the alveolar process, making mesialisation of the second molar into the location of the first molar difficult because the walls of the cortical sinus are too close to the roots of the second molar [10]. With the advent of temporary anchorage, investigating the maxillary sinus has become increasingly important to avoid issues such as sinus perforation and root injury [11]. Therefore, thorough knowledge and anatomical evaluation of the area are essential [7].

In addition, the facial skeleton dimensions and maxillary sinus are closely related. The maxillary sinus has been linked to midfacial growth and contouring, as its shape and dimension reflect the development of bony structures [12]. Alberti [13] assumed that a ‘flat face’ was due to a concave, small frontal wall of the maxillary sinus, whereas a ‘round face’ was due to a convex, larger frontal wall. The maxillary sinus can affect the maxillary position concerning the base of the skull, and the anteroposterior direction of maxillary development can be affected [12]. Some researchers have discovered a strong correlation between mandible length and maxillary sinus dimension [14].

Numerous skeletal differences are observed amongst ethnic groups, particularly between Asian and other ethnic groups, as reported by Algahefi et al. The anteroposterior dimension of the skull in Caucasian subjects is larger than that in Chinese subjects. Except for the anterior sinus index and Sg-N-G angle, which were significantly larger in Chinese patients, the S–N and S–G dimensions and SN G–M angle were statistically significant in Caucasians compared with Chinese patients [15].

Various factors, such as race and malocclusion, could affect the size of the maxillary sinuses [14–17]. According to Shrestha, in skeletal Class II malocclusion, the maxillary sinus is greater than that in skeletal Class III [16]. On the contrary, Yassaei revealed that skeletal Class III malocclusion had larger maxillary sinus dimensions and surface area than the skeletal malocclusion of the other groups [14]. Moreover, Oksayan demonstrated that a hyperdivergent patient has a smaller maxillary sinus than hypodivergent patients [17].

Regarding the size variation of the maxillary sinuses amongst different races, Fernandez mentioned in his study of European and Zulu cadavers that 48.6% of European maxillary sinuses were bigger than Zulu sinuses [18]. However, the measurement of the maxillary sinuses from the cadaveric skull would be inaccurate because of the loss of mucosa and other soft tissues. Consequently, considerable research on maxillary sinuses in living people of different races must be conducted.

Considering that the subject remains unclear, particularly across different races, additional studies must be conducted to determine whether the dimension of the maxillary sinuses varies by race and whether the dimension of the sinuses is related to various skeletal malocclusion. Thus, this study aimed to evaluate maxillary sinus dimensions in patients with various categories of skeletal malocclusion in a sample of Chinese and Yemeni patients to estimate the relationship between maxillary sinus dimensions and skeletal malocclusion and to compare the maxillary sinus surface area and dimensions in these two ethnic groups.

## Methods

### Sample selection

This retrospective study was authorised by the China Medical University Stomatological Hospital’s research ethics committee in Shenyang, Liaoning (No CMUKQ-2022-034). All actions were performed in accordance with the applicable regulations and laws.

Based on a study by Shrestha et al. [16], who examined CBCT analysis of maxillary sinus volume in various craniofacial patterns, the sample size was calculated with an alpha value of 0.05 and a power of 95%. Based on the sample size of this study, 27 subjects were included. The sample size for each skeletal malocclusion group was increased to 30 participants.

In this study 180 cephalic (LC) radiographs were included. The LC was obtained from Chinese participants who were selected retrospectively from orthodontic patients with various types of skeletal malocclusion at China Medical University Stomatology Hospital (Shenyang, China). The LC was taken from Yemeni participants with various types of skeletal malocclusion, who were retrospectively selected from orthodontic patients at Taiz University stomatology Hospital clinics (Taiz, Yemen), and the list of included participants was sent via email.

The following inclusion criteria were applied: (1) no pathological findings in the maxillary sinus; (2) no craniofacial anomalies, no visible facial discord or cleft palate and lip; (3) no prior orthodontic treatment; (4) images for individuals under 18 years ago were excluded from the study because of incomplete sinus development [5]. Cephalometric images displaying alterations in the sinus surface morphology caused by trauma or pathological statuses were excluded from the study. Images with poor quality, which caused difficulty in observing the maxillary sinuses, were excluded.

The individuals were classified into two major groups based on ethnicity: the first group included Chinese patients, whereas the second group included Yemeni patients. Each ethnic group contained 90 patients, 45 men and 45 women, and in each ethnic group, Wits appraisal and ANB angle were used to divide the patients into three groups (0 < ANB ≤ 4, − 1 ≤ Wits ≤ 0) classified as skeletal Class 1, whereas ANB > 4, Wits > 0 is skeletal Class 2, and ANB ≤ 0, Wits <  − 1 is skeletal Class 3. Each group contained 30 patients: 15 men and 15 women.

The dimensions of the sinuses were compared in every ethnic group in accordance with the gender of the subject and skeletal malocclusion classes to determine whether gender subjects with different skeletal malocclusion classes and ethnicity are factors affecting the sinus dimension.

### Maxillary sinus dimensions measurements

**Fig. 1:**
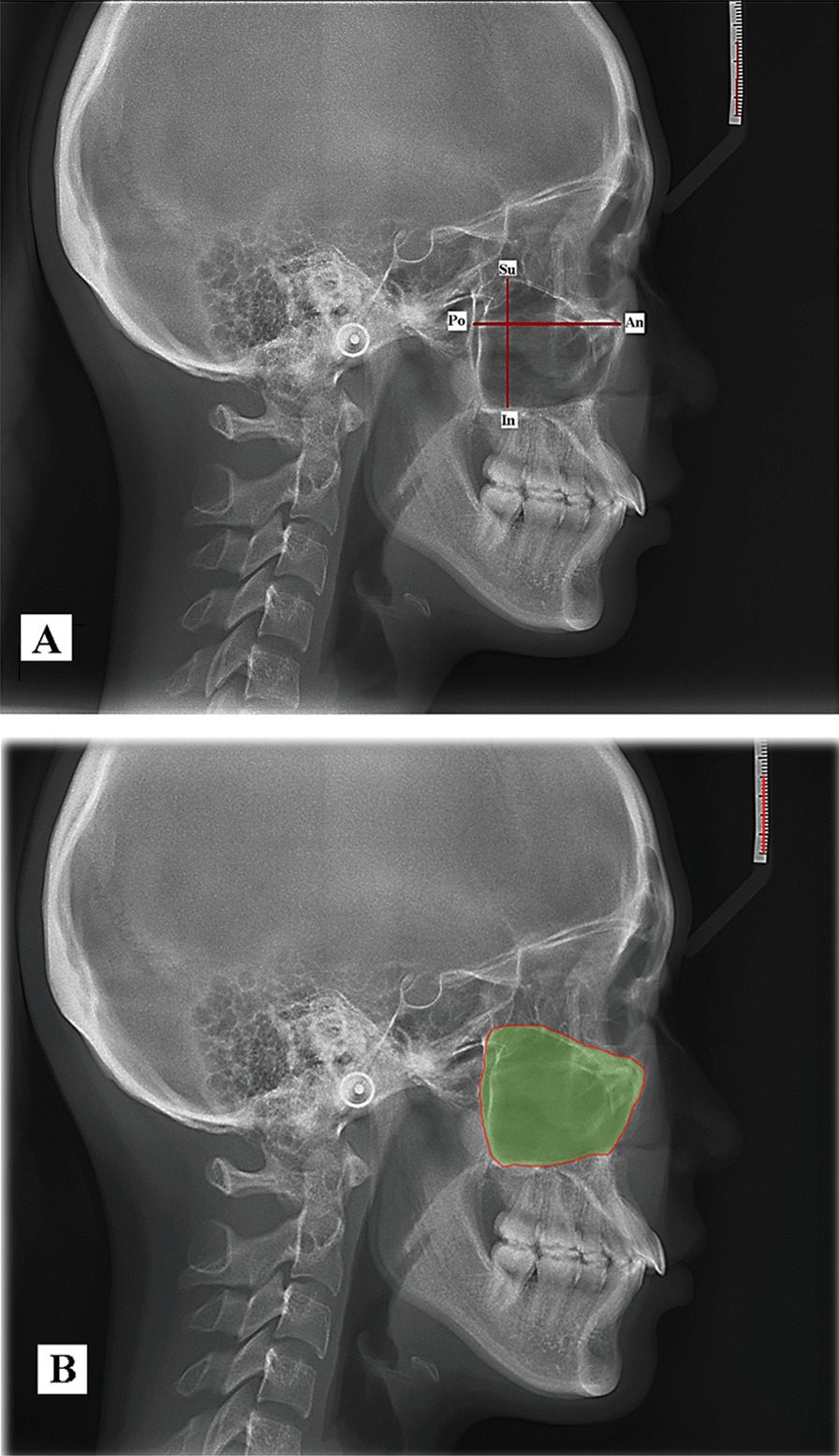
The maxillary sinus indices assessment. **A** Length and height of the maxillary sinus. **B** computed maxillary sinus surface area

The LC was entered using WinCeph 9.0 (Rise Corporation, Sakuragaoka Cho Shibuya Ku, Tokyo, Japan). The right and left sinuses were differentiated, and the left side was correctly traced. The patients were turned to the left when the lateral cephalograms were taken, the left sinus contour was more posterior than the right [3]. The maxillary sinus index was measured as follows [14]: As shown in Fig. 1a, b, (1) a vertical line has been drawn from Su and In to determine the maxillary sinus height (Su refers to the highest point, whereas In refers to the lowest point), and (2) a horizontal line has been drawn from An to Po to define the length of the maxillary sinus (An denotes the frontest point, whereas Po denotes the most backward point). All landmarks and cephalometric measurements used in this study are described in Additional file 1.

In evaluating the significant error of the radiographic measurement, the primary examiner and another observer remeasured randomly selected cephalometric radiographs of 30 patients 3 weeks after the initial measurements. Apart from calculating the percentage of total variation that could be attributed to measurement errors, the mean measurement difference between the primary and secondary measurements for each variable was also calculated.

### Statistical analysis

IBM SPSS Statistics, version 21 for Windows (IBM Corp., Armonk, NY), was used to perform statistical analysis. In determining the reliability and reproducibility of measurements, the intra-class correlation coefficient (ICC) was used. In checking the normal distribution of the data, we used The Shapiro–Wilk test. In comparing mean values amongst groups, we used one-way ANOVA and Tukey’s post hoc test. Data were analysed in accordance with gender, skeletal malocclusion and nationality. The mean and standard deviation for each group were calculated, with the significance level set at P 0.05. The maxillary sinus parameters were evaluated using a simple t-test of total sample data based on nationality and gender groups combined or subdivided separately. Furthermore, we used one-way ANOVA to estimate sinus morphometric data for both malocclusion groups and nationality subgroups. Furthermore, in comparing the mean values amongst the groups, we used the Tukey post hoc test. Pearson’s correlation coefficient was used to calculate the correlation between sinus dimensions and cephalometric indices.

## Results

The reliability of the single and average measurement of maxillary sinus dimensions, surface area and structural parameters with ICC was excellent, ranging between 0.962 and 1.000 (Additional file 2).

The analysed gender data of overall group presented the mean value of the maxillary sinus height (37.58 ± 5.54 mm), length (38.32 ± 4.79 mm) and surface area (1156.08 ± 236.78 mm^2^) in men and the mean value of the maxillary sinus height (33.82 ± 6.05 mm), length (36.04 ± 4.89 mm) and surface area (977.61 ± 253.40 mm^2^) in women. These findings revealed that men had higher maxillary sinus height, length and surface area than women. The differences between men and women are displayed in Table 1.

**Table 1:** The simple t-test results of maxillary sinus dimensions according to gender in the overall, Yemeni, and Chinese group

Variables	Gender				*P* value
	Male		Female		
	Mean	SD	Mean	SD	
*Overall*					
Height	37.5823	5.54856	33.8217	6.05830	0.478
Length	38.3276	4.79078	36.0458	4.89387	0.599
Surface area	1156.0869	236.78255	977.6182	253.40148	0.551
*Yemeni*					
Height	34.0249	4.54864	29.8424	4.98997	0.476
Length	36.2609	4.94877	33.4924	4.64510	0.454
Surface area	1029.6444	214.10170	800.6260	208.87403	0.411
*Chinese*					
Height	41.1398	3.96187	37.8009	4.11730	0.864
Length	40.3942	3.63291	38.5991	3.67857	0.563
Surface area	1282.5293	186.72050	1154.6104	148.92687	0.175

*SD* Standard deviation

Amongst Yemeni, gender shows the mean height (34.02 ± 4.54 mm in men and 29.84 ± 4.98 mm in women), length (36.26 ± 4.94 mm in men and 33.49 ± 4.64 mm in women) and surface area (1029.64 ± 214.10 mm^2^ in men and 800.62 ± 208.87 mm^2^ in women). This results indicate that men have higher mean height, length and surface area than women (Table 1).

Analysis of the dimensions of the maxillary sinus of gender amongst Chinese showed the mean of height (41.13 ± 3.96 mm in men and 37.80 ± 4.11 mm in women), length (40.39 ± in men and 38.59 ± 3.67 mm in women) and surface area (1282.52 ± 186.72 mm^2^ in men and 1154.61 ± 148.92 mm^2^ in women). This result shows that men have more mean height, length and surface area than women. The gender data analysis findings were all statistically insignificant (Table 1). The investigation of the sinus’ surface area, height and length showed an increase in size amongst Chinese compared with those in Yemeni (Table 1).

The malocclusion overall group comparison revealed that skeletal Class II had a more prominent sinus length and surface area increase than Classes I and III, and Classes I and II had an almost comparable height. All these findings were statistically significant, except for maxillary sinus height (Table 2).

**Table 2:** The ANOVA results of maxillary sinus dimensions according to malocclusion in the overall, Yemeni, and Chinese group

Variables	Malocclusions						*P* value
	Class I		Class II		Class III		
	Mean	SD	Mean	SD	Mean	SD	
*Overall group*							
Height	36.6813	5.78130	36.0403	5.52315	34.3843	6.76807	0.103
Length	37.0330	4.76521	38.3802	4.63411	36.1368	5.28401	0.044*
Surface area	1077.44	228.938	1134.64	238.188	989.075	292.48820	0.007*
*Yemeni*							
Height	33.6667	5.39459	33.1507	5.48857	28.9837	3.1425	0.001*
Length	34.5877	4.33291	36.929	4.75	33.0873	5.17106	0.009*
Surface area	968.801	212.352	1011.233	247.042	765.372	185.956	0.001*
*Chinese*							
Height	39.696	4.48269	38.93	3.82906	39.785	4.79291	0.71
Length	39.4783	3.87964	39.8253	4.11927	39.1863	3.29001	0.807
Surface area	1186.08	192.666	1258.05	151.275	1212.78	191.73	0.297

*SD* Standard deviation, *ANOVA* Analysis of variance

*P < 0.05

A comparison of Yemeni malocclusion cases showed that skeletal Class II had a more prominent sinus enlargement in length and surface area than Classes I and III, and the height was nearly equal in skeletal Classes I and II. All these findings were statistically significant (Table 2).

The comparison of malocclusion amongst Chinese individuals indicated no statistically significant difference in the mean value of maxillary sinus height and length amongst all skeletal classes. In addition, the surface area of skeletal Class II was found to be greater than that of Classes I and III. All outcomes were statistically insignificant (Table 2).

The surface area, length and height of the sinus have increased amongst the Chinese compared with those of Yemeni (Table 2).

Regarding the Tukey test results of maxillary sinus dimensions based on malocclusion in the overall group (Table 3), the length of the maxillary sinus was substantially longer in the skeletal Class II group than in the Class III group. Furthermore, the Class II group had the largest maxillary sinus surface area, whereas the Class III group had the smallest (988.4753 ± 292.48820 mm^2^).

**Table 3:** The Post-hoc test (Tukey test) results of maxillary sinus dimensions according to malocclusion in the Overall and Yemeni groups

Variables	Class I/II	Class I/III	Class II/III
*Overall*			
Height	0.831	0.097	0.294
Length	0.293	0.577	0.035*
Surface area	0.438	0.142	0.006*
*Yemeni*			
Height	0.909	0.001*	0.003*
Length	0.144	0.445	0.007*
Surface area	0.729	0.001*	0.001*

*P < 0.05 The difference is statistically significant

Regarding Tukey test results of maxillary sinus dimensions based on malocclusion in the Yemeni group (Table 3), the maxillary sinus height in the skeletal Class II group showed the significantly highest value in Class I without statistically differences with Class II and the lowest in Class III. Furthermore, maxillary sinus length and surface area were substantially higher in Class II and lower in Class III.

Analysis of surface area, length and height of the overall ethnicity revealed that Chinese individuals have larger maxillary sinuses than Yemeni. Chinese had the biggest sinuses, with an average height of 39.47 ± 4.35 mm, a length of 39.49 ± 3.74 mm and a surface area of 1218.56 ± 179.82 mm^2^. By contrast, Yemeni individuals had the smallest maxillary sinus size, with an average height of 31.93 ± 5.19 mm, 34.87 ± 4.97 mm and a surface area measurement of 915.13 ± 239.77 mm^2^. The mean and standard deviation values for ethnicity are presented in Table 4. The outcomes of the maxillary sinus analysis were as follows: the difference in surface area between Chinese and Yemeni sinuses was highly significant, with Chinese sinuses having a substantially higher surface area than Yemeni sinuses (*P* = 0.000).

**Table 4:** The simple t-test results of maxillary sinus dimensions according to the overall group of ethnicities

Variables	Overall group of ethnicities				*P* value
	Yemeni		Chinese		
	Mean	SD	Mean	SD	
Height	31.9337	5.19242	39.4703	4.35422	0.185
Length	34.8767	4.97116	39.4967	3.74559	0.094
Surface area	915.135	239.773	1218.57	179.828	0.050*
Valid N (listwise)	90		90		

*SD* Standard deviation

*P < 0.05

Table 5 shows that the dimension and surface area of the maxillary sinus displayed a significant positive relationship with the SNA and SNB angles. The maxillary length (Co–A) was positively correlated with the maxillary sinus dimension and surface area. By contrast, the mandibular length (Co–Gn) was positively correlated with the maxillary sinus height. Furthermore, a negative correlation was observed between the maxillary sinus dimension and surface area, as well as the gonial and GoGn–Sn angles. Furthermore, a significantly positive correlation was observed between NA–APO and NA–FH angles and maxillary sinus surface area.

**Table 5:** The correlation between dimension, maxillary sinus surface area, and skeletal parameters in the overall group

Skeletal parameters	Correlation	Height	Length	Surface area
SNA	Pearson correlation	0.411**	0.330**	0.436**
	Sig. (2-tailed)	0.000	0.000	0.000
SNB	Pearson correlation	0.368**	0.241**	0.313**
	Sig. (2-tailed)	0.000	0.001	0.000
Co–A	Pearson correlation	0.156*	0.175*	0.203**
	Sig. (2-tailed)	0.036	0.019	0.006
Co–Gn	Pearson correlation	0.173*	0.017	0.084
	Sig. (2-tailed)	0.020	0.820	0.265
GoGn–SN	Pearson correlation	− 0.400**	− 0.272**	− 0.363**
	Sig. (2-tailed)	0.000	0.000	0.000
Gonial angle	Pearson correlation	− 0.220**	− 0.168*	− 0.222**
	Sig. (2-tailed)	0.003	0.024	0.003
NA–FH	Pearson correlation	0.118	0.104	0.201**
	Sig. (2-tailed)	0.116	0.166	0.007
NA–APO	Pearson correlation	0.046	0.110	0.165*
	Sig. (2-tailed)	0.537	0.143	0.026

*Correlation is significant at the 0.05 level (2-tailed)

**Correlation is significant at the 0.01 level (2-tailed)

## Discussion

The lateral cephalogram has become an essential orthodontic record frequently used for effective diagnosis and treatment planning [19]. Malocclusion has been defined as undesirable variations from the normal, and the morphologic aspects of malocclusion have been widely explored using lateral cephalogram analysis [20].

A lateral cephalogram shows several anatomical features that can be used to assess malocclusion. The maxillary sinuses are anatomical landmarks that can be easily analysed, and they do not present duplicate data in a lateral cephalogram radiograph [21].

The maxillary sinuses are the major paranasal sinuses and are the earliest to develop in intrauterine life [22]. They are connected to the pterygomaxillary and infratemporal fossa, and they have a pyramidal form [23]. The maxillary alveolar process forms the floor of the sinus. It is also anatomically and functionally related to posterior maxillary teeth [24].

In general, the maxillary sinuses have several purposes. Rae et al. [25] characterised the function of the maxillary sinus as a respiratory function, thermoregulation and trauma protection. They occupy considerable cranial space, and they have been the subject of research into their function and the factors influencing their shape and size.

### Dimension analysis of the maxillary sinus

Maxillary sinus dimensions change with gender. Based on the findings of the present study, men and women have varying maxillary sinus diameters. This difference is similar to that of other studies [26–28], which considered gender factors. Considering that men showed greater height, length and surface area than women, they had mentioned two possible interpretations. Firstly, according to Enlow [29], men require a larger lung to support their substantially larger body organs and muscles. Second, men required a significant airway that began at the nose and extended to the nasopharynx. In another way, the physiological changes and structure of the nasal cavity resulted from respiratory-related requirements, such as humidification and warming of breathed air. In addition, the maxillary sinus increases in size because of the filling of remnant space inside the nasomaxillary complex. The maxillary sinus surface area of men is related to body height and weight in the international literature reported by Ariji et al. [30], which may explain why men’s sinus dimensions rise more than women.

The current study shows the mean value of maxillary sinus amongst Yemeni of different gender in accordance with height (34.02 ± 4.54 mm in men and 29.84 ± 4.98 mm in women), length (36.26 ± 4.94 mm in men and 33.49 ± 4.64 mm in women) and surface area (1029.64 ± 214.10 mm^2^ in men and 800.62 ± 208.87 mm^2^ in women) and amongst Chinese of different gender in accordance with height (41.13 ± 3.96 mm in men and 37.80 ± 4.11 mm in women), length (40.39 ± 3.63 mm in men and 38.59 ± 3.67 mm in women) and surface area (1282.52 ± 186.72 mm^2^ in men and 1154.61 ± 148.92 mm^2^ in women). This result has shown statically non-significant difference, as shown in the tables of results. These results are consistent with those of previous studies conducted by Sharma et al. [31]and Uthman et al. [26]. However, other studies showed results lower than these measures [27]. Furthermore, other studies [28, 32] have reported wider and higher maxillary sinuses than our results. However, a survey by Uchida et al. [33] used the cadaver skull and reported no statistically significant difference regarding sides, age or gender.

### Changes in maxillary sinus dimensions with malocclusions

The maxillary sinus dimensions of various skeletal malocclusions were measured in this study. Our findings in the overall group revealed that the skeletal Class II group had significantly larger dimensions than the Classes I and III groups. Except for height, which appeared almost equal in the two groups, skeletal classes I and II were not statistically significant.

In addition, amongst Yemenis, the results showed a significant statistical difference in mean sinus’s height, length and surface area in skeletal Class II malocclusion. However, sinus height was greater in the skeletal Class I group. For Chinese, the surface area in the skeletal Class II group was greater than that in the rest of the groups, with an approximately equal height and length in all malocclusion groups. These variations could be due to the use of a two-dimensional lateral cephalogram, a small number of samples, ethnic differences in the samples and the age of the samples utilised in our study not being standardised.

All the findings in the current study revealed that the maxillary sinus diameter was more significant in skeletal Class II than in Classes I and III. Thus, other studies [16, 34–36] considered malocclusion factors. Dibbets et al. [37] and Hopkin et al. [38] interpreted these results as they concluded in their research that men have a bigger cranial base than women, and individuals with skeletal Class II malocclusion have a bigger cranial base than those with Class I or Class III malocclusion. Patients with bigger cranial bases usually have larger maxillary sinuses. Consequently, he suggests that the mean value of male skeletal Class II malocclusion is the highest. These results were in accordance with the ones found in this study.

### Changes in maxillary sinus dimensions with ethnicities

In the current study, the maxillary sinus dimensions did not exhibit a static significance with ethnicities, except for the surface area of the sinus, which showed statistical significance (*P* < 0.05). This might result from the small sample sizes within each ethnic group. However, the sinus surface area showed a significant increase. Based on our findings, the dimensions of the maxillary sinuses differ from ethnicity to ethnicity, as the dimensions of the sinus were larger in Chinese than in Yemeni. These differences may be due to the ethnic differences found by Rhee et al. [39]. When they compared Caucasian and East Asian people’s attractive faces, particularly midfacial width measurements, they found that the width and height of the middle of the face in East Asian people were greater than those of Caucasians. This conclusion is consistent with previous studies performed on people of different races and geographic regions [18, 40].

Notably, these factors could differ from the maxillary sinus surface area values. As shown by Kawarai et al. in a study of computed tomography scans of Japanese ancestry, the MSV was greater in Japanese than in people of other races (mean right MSV: 23.6 cm^3^, mean left MSV: 20.9 cm^3^). This outcome was linked to variances in this ethnicity’s height–weight ratio and differences in volume measurement methods [40]. In addition, Fernandez conducted research on cadavers from Europe and Zulu of different ethnicities and gender and concluded that the MSV of the two races differed statistically [18].

Regarding the association amongst dimension, maxillary sinus surface area and skeletal parameters, the current findings revealed a substantial relationship between maxillary sinus length, height, surface area, SNA and SNB angles. The maxillary sinus dimensions increased with the increase of SNA and SNB angles. Endo et al. [3] reported the same conclusion.

In addition, the maxillary length was related to sinus height, length and surface area. Therefore, a greater maxillary length is associated with an increase in sinus height, length and surface area, which can be explained by the increased midface length associated with relative prognathic maxillary cases.

Moreover, sinus height had a significant relationship with the length of the mandible, and sinus height was higher amongst patients with a longer mandible, which could be explained by the considerable mandible length linked with the retrognathic maxilla and prognathic mandible situations, as shown in skeletal Class III skeletal malocclusion.

The current study found a relationship amongst NA–APOg, NA–FH angle and the maxillary sinus surface area, indicating a tendency for maxillary prognathism in patients with a greater maxillary sinus surface area. Meanwhile, the dimension and surface area of the sinus have a considerable inverse relationship with the gonial and GoGn–Sn angles, with the dimension and surface area of the sinus decreasing as the degree of these angles increases.

These findings have various dental implications. Orthodontic movement of teeth in the posterior maxillary area requires special consideration when the maxillary sinus is large, as in males, and skeletal Class II skeletal malocclusion. Based on case studies by Park et al. [41] and oh et al. [42], closing the spaces caused by the loss of the posterior maxillary teeth through the maxillary sinus is complex. In obtaining a positive result, modest forces must be used to increase treatment duration. When the posterior root apices protrude into the maxillary sinus, intrusion of teeth can be difficult and slow, and extremely little force is necessary [9, 43]. Likewise, insertion of TADs in the maxillary posterior buccal areas also requires special consideration. In such cases, orthodontic mini-plates may be used as an alternative to mini-implants [44].

## Limitation

This study is the first to focus on the relationship between skeletal malocclusion and changes in the dimensions of the maxillary sinus between Yemeni and Chinese. However, some limitations are still found in this study, such as the small sample size in addition to the 2D imaging method instead of 3D imaging. The use of three-dimensional techniques to improve parameter diagnostics and assessments is suggested in future studies. Only two ethnic groups were included in this study: studies with a larger sample size and diverse ethnicities.

## Conclusion

1. In both ethnic groups, men sinuses were larger than women, and Chinese had larger maxillary sinuses than Yemeni.
2. Maxillary sinus dimensions were larger in skeletal Class II malocclusions than in other groups of both ethnicities.
3. The SNA, SNB and Co–A showed a strong positive correlation with maxillary sinus size and surface area.
4. A significant link was observed between Co–Gn linear and maxillary sinus length. Furthermore, the NA–APO and NA–FH angles were significantly related to the surface area of the sinus.
5. The gonial and GoGn–Sn angles negatively correlate with maxillary sinus dimensions and surface area.

## Supplementary Information

**Additional file 1.** Landmarks and description of the cephalometric measurements used in this study.

**Additional file 2.** Reliability beetween dimension, maxillary sinus surface area, and skeletal parameters in the overall group.

## Data Availability

Due to (ownership of data), the datasets created and analyzed during the current study are not publicly accessible, but they are available from the corresponding author upon reasonable demand. All data and materials were available at the clinics of the Stomatology Hospital, China Medical University, Liaoning, China, and Taiz University stomatology Hospital clinics (Taiz, Yemen).

## Abbreviations

TADs: Temporary anchorage devices
LC: Lateral cephalometric
2-D: Two-dimensional
3-D: Three-dimensional
MSV: Maxillary sinus volume
ICC: Intra-class correlation coefficient
