# Supplementary material for: A comparison of maxillary sinus diameters in Chinese and Yemeni patients with skeletal malocclusion
Source: BMC Oral Health. 2022 Dec 9;22:582. doi: 10.1186/s12903-022-02633-0 (PMC9733360; doi:10.1186/s12903-022-02633-0)
Supplement: Supplementary file 1 — Additional file 1. Landmarks and description of the cephalometric measurements used in this study. [file 12903_2022_2633_MOESM1_ESM.docx]

Additional file 1 Landmarks and description of the cephalometric measurements used in this study

| **Name** | **Definition** |  |
| --- | --- | --- |
| **Landmarks** | | |
| **Sella (S)** | The center of Sella. | |
| **Nasion (N)** | The highly anterior point of the frontonasal suture. | |
| **Subspinale (A)** | The lowest point on the outer contour of the maxillary alveolar process. | |
| **Submentale (B)** | The lowest point on the outer contour of the mandibular alveolar process. | |
| **Orbitale (Or)** | Lowest point of the bony orbit. | |
| **Porion (Po)** | Highest point of the external auditory meatus. | |
| **Pognion (Pog)** | The most anterior point of the symphysis. | |
| **Gnathion (Gn)** | The most anterior inferior point on the bony chin. | |
| **Mention (Me)** | The most inferior point of the outline of the symphysis. | |
| **Gonion (Go)** | The most posterior inferior on the angle of mandible. | |
| **Articular (Ar)** | The point on the ramus's posterior border where it meets the basilar part of the occipital bone. | |
| **Condylion (Co)** | Most posterior and superior points on the mandibular condyle. | |
| **Measurements** |  | |
| **SNA (º)** | Anteroposterior position of A‑point. | |
| **SNB (º)** | Anteroposterior position of B‑point. | |
| **ANB (º)** | Anteroposterior relationship between A‑point. | |
| **Wits (mm)** | Distance between a line drawn perpendicular to the occlusal plane from Point A and a line drawn perpendicular to the occlusal plane from Point B. | |
| **Angle of convexity (º)** | Angle between NA line and APog. | |
| **Mandibular plane angle(º)** | The angle among the anterior cranial base and the GoGn. | |
| **Mandibular length (mm)** | Distance between points Co and Gn. | |
| **Gonial Angle (º)** | The angle formed between the mandibular plane and a tangent to the posterior border of the mandible passing through Articular. | |
| **Maxillary depth angle (º)** | Angle formed between NA and FH planes. | |
| **Maxillary length (mm)** | Distance between points Co and A. | |
