# Supplementary material for: A comparison of maxillary sinus diameters in Chinese and Yemeni patients with skeletal malocclusion
Source: BMC Oral Health. 2022 Dec 9;22:582. doi: 10.1186/s12903-022-02633-0 (PMC9733360; doi:10.1186/s12903-022-02633-0)
Supplement: Supplementary file 2 — Additional file 2. Reliability beetween dimension, maxillary sinus surface area, and skeletal parameters in the overall group. [file 12903_2022_2633_MOESM2_ESM.docx]

Additional file 2 Reliability beetween dimension, maxillary sinus surface area, and skeletal parameters in the overall group.

|  | **ICC** |  |
| --- | --- | --- |
| **VARIABLES** | **Single measure** | **Average measure** |
| **skeletal parameters** |  |  |
| **SNA** | 0.992 | 0.996 |
| **SNB** | 0.990 | 0.995 |
| **Co-A** | 0.999 | 0.999 |
| **Co-Gn** | 0.999 | 1.000 |
| **GoGn-SN** | 0.998 | 0.999 |
| **Gonial Angle** | 0.999 | 1.000 |
| **NA-FH** | 0.962 | 0.980 |
| **NA-APO** | 0.998 | 0.999 |
| **maxillary sinus dimensions** |  |  |
| **Height** | 0.998 | 0.999 |
| **Length** | 0.999 | 1.000 |
| **Surface Area** | 1.000 | 1.000 |

* ICC: intra-class correlation coefficient
